# Supplementary material for: The impacts of project uncertainty and relationship conflict on relationship continuity in construction: The role of political skill
Source: PLoS One. 2025 Nov 13;20(11):e0334555. doi: 10.1371/journal.pone.0334555 (PMC12614530; doi:10.1371/journal.pone.0334555)
Supplement: Attachment — Submitted filename: Response to Reviewers.doc [file pone.0334555.s003.doc]

**Dear editors and reviewers:**

Thank you for reviewer’s comments and suggestions which are relevant for us. Those comments are all valuable and very helpful for revising and improving our paper, as well as an important guiding to our research. According to the comments, we have made a significant revision. The changes have been highlighted within the 'Revised Manuscript with Track Changes' by using the track changes mode in MS Word. We also upload a separate file labeled 'Manuscript'.

The comments and responses as follows:

**Reviewer #1:**

**This paper investigates how project uncertainty and relationship conflict influence the continuity of relationships in the construction industry. Given the complex, dynamic nature of construction projects, understanding how these factors affect long-term collaborations is crucial for enhancing project success and fostering sustainable relationships between stakeholders.**

**1. One of the main weaknesses of the paper is the lack of a strong, detailed conceptual framework. While the relationship between project uncertainty, conflict, and relationship continuity is established, the underlying theories and mechanisms that connect these concepts are not fully explored. A more robust theoretical grounding could help clarify how project uncertainty and conflict lead to disruptions in relationships and why these disruptions occur in certain contexts but not others.**

***Response:*** *Thank you for reviewers careful review. According to reviewer’s good suggestion, we have carefully revised the paper. Firstly, in order to make the conceptual framework clearer, based on transaction cost theory（Line 153），self-classification theory（Line 171）, social cognition theory（Line 197） and contingency theory（Line 243）, the paper reorganizes and analyzes the variables and the relationship between variables. Secondly, the manuscript rearranges the research progress, increases the research gap and the problems to be solved in this research. Especially in the last paragraph of the introduction, the problems to be solved in this study are clarified one by one.*

**2. The paper provides valuable insights into the theoretical aspects of project uncertainty and relationship conflict, but it falls short in offering practical recommendations for construction managers or stakeholders. For example, practical strategies like fostering open communication, establishing clearer contractual terms, or implementing conflict resolution frameworks would significantly enhance the paper’s applicability to real-world situations.**

***Response:*** *Thank you for reviewer’s good suggestion. Through the research of this thesis, project managers should have a correct understanding of project uncertainties and realize that project uncertainties can affect the long - term cooperative relationships among stakeholders through relationship conflicts. Previous studies have introduced many formal governance mechanisms for maintaining the cooperation of project stakeholders (such as contractual constraints, regular meetings, etc.). This research proposes the role of flexible factors in project cooperation. Politically skilled project partners view the relationship conflicts caused by project uncertainty as opportunities and seek to take a positive approach to the task of resolving uncertain problems. Some suggestions have been made to improve and enhance individuals’ political skills in project management. For instance, project human resources and organizational development departments should view political skill as an important person ability that is related to the recruitment process as well as promotion and project team building. On the other hand, some political skill training programs should be designed and developed for all project participants. It is believed that these practical suggestions can serve as a reference for project managers in their project management.*

**Reviewer #2:**

**The paper entitled” The impacts of project uncertainty and relationship conflict on relationship continuity in construction: The role of political skill” deals with a very interesting topic. In general, I appreciate the aims of this work; it is quite interesting and informative to most readers of this field. However, I have the following comments that hopefully help the authors improve their paper:**

**1. Regarding the literature review, I strongly recommend that the authors include a summary table of their comprehensive literature review. Such a table would not only help identify gaps in the existing literature but also enhance the contribution of this work by providing a clear overview of the relevant research in the field.**

***Response:*** *Thank you for reviewers careful review. As the reviewers said, listing a literature review table does have a very good effect on classifying the literature of this research and providing a clear overview of the relevant research in the field. Nevertheless, in order to present the logical relationships and hypotheses between variables one by one, numerous literatures are repeatedly cited in the literature review section. Therefore, this paper does not list the references in summary table. secondly, in a previous study by my team members, a detailed literature review of “project uncertainty” was conducted and presented in table form (Antecedents of relationship conflict in cross-functional project teams. Project Management Journal, 2016, 47(5): 52-69.). Thirdly, in order to make the conceptual framework clearer, based on transaction cost theory（Line 153），self-classification theory（Line 171）, social cognition theory（Line 197） and contingency theory（Line 243）, the paper reorganizes and analyzes the relationship between variables. It is hoped that through the above - mentioned revisions, the paper can present* a clear overview of the relevant research in this field.

**2. As usual, thorough proof-reading is recommended. I wish the author(s) all the best for their research and that these comments will be useful to them in improving the paper.
*Response:*** *According to the reviewers' suggestions, this paper has gone through the proof - reading process. This paper has improved in terms of expression and readability.*
