# Supplementary material for: The impacts of project uncertainty and relationship conflict on relationship continuity in construction: The role of political skill
Source: PLoS One. 2025 Nov 13;20(11):e0334555. doi: 10.1371/journal.pone.0334555 (PMC12614530; doi:10.1371/journal.pone.0334555)
Supplement: Attachment — Submitted filename: Response_to_Reviewers_auresp_2.doc [file pone.0334555.s004.doc]

**Dear editors:**

Thank you for reviewer’s comments and suggestions which are relevant for us. Those comments are all valuable and very helpful for revising and improving our paper, as well as an important guiding to our research. According to the comments, we have made a significant revision. The changes have been highlighted within the ‘Revised Manuscript with Track Changes’ by using the track changes mode in MS Word. We also upload a separate file labeled ‘Manuscript’.

The comments and responses as follows:

1. Please review your reference list to ensure that it is complete and correct. If you have cited papers that have been retracted, please include the rationale for doing so in the manuscript text, or remove these references and replace them with relevant current references. Any changes to the reference list should be mentioned in the rebuttal letter that accompanies your revised manuscript. If you need to cite a retracted article, indicate the article’s retracted status in the References list and also include a citation and full reference for the retraction notice.

**Response:**

We have reviewed the reference list to ensure that it is complete and correct. The reference list have not cited papers that have been retracted.

2. First and foremost: The linguistic accuracy and professional tone of the manuscript. To ensure that your research conveys its findings with the utmost clarity and professionalism, I sincerely recommend that the manuscript undergoes copyediting by a specialized agency.

**Response:**

We accepted editors suggestions regarding the language problem. We have already had the entire text linguistically polished by a professional institution (AJE) to ensure that our research conveys its findings with the utmost clarity and professionalism.

3. The discussion and conclusion section needs to be more nuanced and analytical. The authors should critically evaluate their findings, comparing them with existing literature and addressing any discrepancies or inconsistencies.
**Response:**

We fully agree with this suggestion. Following the suggestion, we have added a large amount of analysis to the conclusion section. The specific revisions can be seen in the “Revised Manuscript with Track Changes”
