# Supplementary material for: The impacts of project uncertainty and relationship conflict on relationship continuity in construction: The role of political skill
Source: PLoS One. 2025 Nov 13;20(11):e0334555. doi: 10.1371/journal.pone.0334555 (PMC12614530; doi:10.1371/journal.pone.0334555)
Supplement: Attachment — Submitted filename: Response_to_Reviewers_auresp_3.doc [file pone.0334555.s005.doc]

**Dear reviewers:**

Thank you for reviewer’s comments and suggestions which are relevant for us. Those comments are all valuable and very helpful for revising and improving our paper, as well as an important guiding to our research. According to the comments, we have made a significant revision. The changes have been highlighted within the “Revised Manuscript with Track Changes”. We also upload a separate file labeled “Manuscript”.

The comments and responses as follows:

**Reviewer #3: Accept the manuscript. All the reviews were addressed in the former revision. I with good luck with your research.**

**Response:** I sincerely thank you for thoroughly reviewing my manuscript and recommending its acceptance.

**Reviewer #4: The manuscript titled “The impacts of project uncertainty and relationship conflict on relationship continuity in construction: The role of political skill” presents an investigation into the relational dynamics within construction projects, focusing on project uncertainty and political skill. While the topic is timely and potentially valuable for the construction management field, the paper suffers from several foundational and structural deficiencies that substantially undermine its academic rigor and suitability for publication in its current form.

One of the most critical issues in this study is the lack of a clearly established theoretical foundation. Although the manuscript briefly references the contingency theory in relation to political skill, it fails to adopt any managerial or organizational theory as a grounding framework for its conceptual model or hypotheses. This omission severely limits the paper's ability to demonstrate theoretical contribution, and the study appears to be constructed without anchoring its variables or assumptions in any established theoretical discourse. The hypotheses are presented in isolation, disconnected from a guiding framework that would validate their inclusion and enhance their interpretive power.**

**Response:** We sincerely thank the reviewer for this profound and constructive feedback. A clear theoretical foundation is crucial for enhancing the explanatory power and academic value of the research. In response to this valuable suggestion, we have refined the revised manuscript by explicitly adopting and elaborating on the “Contingency Theory” as the core theoretical framework of our entire study. (1) Strengthening the theoretical framework statement in the introduction: We have added a new paragraph in the introduction stating that this study is grounded in Contingency Theory. We pointed out that “Based on the contingency theory, there is no universally applicable optimal management method for organizations; the effectiveness of a management approach depends on the degree of alignment between management practices and the external environment (such as uncertainty). Guided by this principle, this research focuses on political skill as a contingency factor to examine the ways in which it mitigates the negative impact of project uncertainty on partner relationship continuity.” (Lines 112-117) This provides a clear theoretical perspective for the entire study. (2)In the literature review and hypothesis development section, we introduce political skill, defining it as a crucial “contingency-coping mechanism”. We elaborate on how individuals with high political skill leverage their social insight and interpersonal influence to effectively manage interactions, mediate disagreements, and rebuild trust in highly uncertain and conflict-ridden environments, thereby mitigating the negative impacts of uncertainty(Lines 251-253). Additionally, in the hypothesis development section, we also employed Transaction Cost Theory (Line 159), Information Processing Theory (Line 173), and Self-Categorization Theory (Line 177). This significantly strengthened the theoretical logic and persuasiveness of our hypothesis formulation. (3) In the discussion section, we closely aligned our findings with Contingency Theory to explain their theoretical implications: When discussing the research results, we particularly emphasized interpreting their theoretical significance from the perspective of Contingency Theory. Our findings not only validate the relationships between variables but, more importantly, reveal how “political skill” as a crucial individual trait, achieves “Person-Environment Fit” to address uncertainty as an environmental contingency factor. This significantly enhances the theoretical depth and contribution of our research conclusions.

Through these revisions, we hope the reviewers will recognize that we have successfully deeply integrated contingency theory as the “backbone” of our research, providing a much-needed theoretical anchor for the conceptual model and hypotheses. We believe the theoretical rigor and contribution of the revised manuscript have been substantially enhanced.

**The abstract is another area of major concern. It is poorly written and unstructured, lacking the conventional components expected in a scholarly summary. It fails to articulate the study’s aim, theoretical grounding, methodology, major findings, and implications in a coherent sequence. The use of informal language, including the repetitive use of "we," further detracts from its academic tone. Additionally, the abstract’s formatting is inconsistent and reflects a lack of attention to publication standards, which compromises the professionalism of the presentation.**

**Response:** We sincerely thank the reviewers for their constructive feedback regarding the abstract. We acknowledge that the previous version was inadequately structured and written in an informal tone, which failed to meet academic standards. We will thoroughly revise the abstract to include all conventional components: clearly stating the research objectives, theoretical foundation, methodology, key findings, and implications. In addition, we will ensure a coherent flow of ideas, replace informal expressions with appropriate academic language, and eliminate repetitive use of first-person pronouns. Formatting issues will be corrected in accordance with target journal guidelines. We appreciate these helpful comments and believe the revised abstract will significantly improve in clarity and professionalism.

**Methodologically, the paper exhibits weaknesses that raise significant questions about the validity and generalizability of the findings. The sample size of 230 participants is inadequately justified, particularly given the context of the expansive Chinese construction industry. There is no explanation of the overall population size or statistical rationale for selecting this number of respondents. Moreover, essential contextual details-such as the specific cities where the data was collected, the type of construction sectors surveyed, and the rationale for choosing these projects-are conspicuously absent. This lack of clarity obstructs the reader's ability to understand the representativeness of the sample and the relevance of the findings.**

**Response:** We sincerely thank the reviewer for raising these important methodological points. We agree that greater clarity and justification are necessary regarding the sample and data collection context. In response to these concerns, we will provide the following revisions in the revised manuscript: (1) We will include a more thorough justification for the sample size of 230, with reference to similar studies in the field and power analysis where applicable, to clarify its adequacy for the analyses performed. (2) We will specify the overall target population size and sampling framework (in Line 349-351) to enhance transparency regarding sample selection and representatives. (3) The criteria for selecting specific projects and participants are definitive (in Line 339-340). This will help readers better assess the generalization and relevance of the findings.

**The manuscript also glosses over potential bias issues. While there is a brief mention of common method bias and two techniques are cited for its evaluation, the discussion is vague and lacks methodological depth. The treatment of common method variance is presented as a checklist rather than a critical analysis, which weakens the study’s internal validity. Furthermore, there is no substantial elaboration on how the data collection procedures addressed potential threats related to self-reported survey designs.**
 **Response:** We sincerely thank the reviewers for their constructive feedback regarding the common method variance. Regarding common method bias (CMB), a two-step procedure was adopted to test for potential common method bias. First, a series of single factor analyses are conducted on each constructed metric, and there is no single factor that can explain the covariance. In addition, Whitney’s (2001) marker variable method was applied. These validation procedures are also commonly used in academic research. We believe these revisions will significantly improve the methodological rigor of our paper and provide readers with a transparent assessment of potential biases.

**Equally problematic is the discussion section, which falls short of providing critical engagement with existing literature. The authors do not compare their findings with recent or relevant studies, nor do they contextualize their contributions within broader research debates in project management or organizational behavior. The absence of such comparative discussion limits the scholarly impact of the paper and hinders readers from appreciating its novelty or practical significance. The results are presented descriptively, but without analytical depth or engagement with recent empirical evidence from similar settings.**
 **Response:** We sincerely thank the reviewer for this critical feedback regarding the discussion section. We acknowledge that our original discussion lacked sufficient comparative analysis with recent literature and deeper engagement with broader scholarly debates, which limited the articulation of our study’s contributions. In response to this comment, we will thoroughly revise the discussion section to achieve the following: (1) We will contextualize the contributions of our results within ongoing research debates-particularly those related to political skill, and uncertainty in construction projects-to better highlight the theoretical and practical significance of our work. (2) We will strengthen the analytical depth of the discussion to interpret their implications in light of existing empirical and theoretical literature. We believe these revisions will significantly enhance the scholarly impact of the paper and allow readers to more fully appreciate the novelty and relevance of our findings.

**In terms of academic writing quality, the overuse of first-person pronouns and informal phrasing detracts from the professionalism of the paper. Although the manuscript claims to have undergone linguistic editing, grammatical errors and syntactical issues remain prevalent throughout the text. These issues affect the clarity and precision of the arguments, making it difficult to follow the line of reasoning in several key sections.**

**Response:** We thank the reviewer for highlighting these important issues concerning the academic writing quality of our manuscript. We sincerely apologize for the shortcomings in language and style, which have impacted the clarity and professionalism of the paper. In response to this feedback, we will undertake a comprehensive revision of the entire manuscript to address these concerns. Specifically, we will: (1) Significantly reduce the use of first-person pronouns and replace them with more formal and objective academic phrasing where appropriate. (2) Carefully review and correct grammatical errors, syntactical inaccuracies, and informal expressions throughout the text. (3) Ensure that sentence structures are clear and precise to strengthen the logical flow and readability of the arguments. We will also seek additional professional language editing services to ensure the revised manuscript meets the high linguistic standards expected for publication. We appreciate the reviewer’s careful reading and constructive suggestions, which will greatly improve the overall quality of our paper.

**Overall, the manuscript exhibits substantial theoretical, methodological, and structural deficiencies. Its lack of a guiding theoretical lens, weak methodological justification, incomplete contextual information, insufficient analytical discussion, and problematic academic tone collectively render it unsuitable for publication in its current form. These issues are not minor or isolated, but systemic across the paper, suggesting the need for significant redevelopment to meet scholarly standards.**

**Response:** We are dedicated to addressing each of the points raised above and believe that these revisions will significantly improve the quality and suitability of the manuscript for publication. We greatly value the opportunity to revise and resubmit a substantially improved version and thank the reviewers again for their invaluable guidance.

**Reviewer #5: Thank you for the opportunity to review the revised manuscript on the relationship between project uncertainty and relationship continuity mediated by relationship conflict and moderated by political skills.
 While the authors have made improvements based on the reviewers' suggestions, several key areas still require attention to make the manuscript publishable:**

**1. The introduction needs to more effectively establish the current state of research and the study's unique contribution. Additionally, the introduction of political skills should be more nuanced, acknowledging its potential negative impacts ("double-edged sword" nature) rather than presenting it solely in a positive light.**

**Response:** We sincerely thank you for this insightful suggestion. We have revised the Introduction section to better contextualize the current research landscape and more clearly articulate the unique contributions of our study. Additionally, we have incorporated a more balanced view of political skill, acknowledging its potential negative implications where relevant. Please see lines 83-87 and 102-105 in the revised manuscript.

**2. A citation for transaction cost theory is missing.**

**Response:** We apologize for this oversight. We have now included a citation for Transaction Cost Theory (Williamson, 1985) in the section where it is first mentioned. Please see line 156.

**3. Some citations (e.g., lines 72, 75) need to be corrected. An error is also noted on line 194.**

**Response:** Thank you for pointing this out. We have carefully reviewed and corrected all citations throughout the manuscript.

**4. Lines 380-384 contain confusing information regarding the variables and instruments used for data collection.**

**Response:** We have rewritten this section to clarify the data collection process and the variables measured. We now more clearly distinguish between the constructs and their operationalization. Please see the revised Methodology section.

**5. The discussion section is primarily descriptive and disconnected from the existing literature, with the exception of two citations. It should be expanded to more thoroughly connect the findings to previous studies rather than relying on intuition.**

**Response:** We agree completely. We have substantially revised the Discussion section to integrate more references to prior literature and to better contextualize our findings within existing theoretical frameworks. We now explicitly compare and contrast our results with those of previous studies. Please see the revised Discussion.

**6. The manuscript still contains numerous typos and awkward sentences (e.g., lines 66, 111, etc.). In Table 2, "Soble" should be corrected to "Sobel".**

**Response:** We have thoroughly proofread the manuscript to correct typos and improve sentence fluency. “Soble” in Table 2 has been corrected to “Sobel.” We also thank you for pointing out specific lines—these have been addressed.

**7. The meaning of abbreviations like EPC or Hydra must be defined for the reader.**

**Response:** We have now defined all abbreviations upon first use. For example: EPC (Engineering, Procurement, and Construction) (Line 498); Hydra: Referenced from Greek mythology, meaning a multi-headed serpent; used here metaphorically to illustrate the persistent and multiplying nature of uncertainty (Line 487-489).

**8. The tables are also difficult to interpret and follow.**

**Response:** We have reformatted all tables to improve clarity and readability. We have also added more descriptive titles and notes to aid interpretation.
